# Large cell neuroendocrine lung carcinoma: consensus statement from The British Thoracic Oncology Group and the Association of Pulmonary Pathologists

**DOI:** 10.1038/s41416-021-01407-9

**Published:** 2021-09-06

**Authors:** Colin R. Lindsay, Emily C. Shaw, David A. Moore, Doris Rassl, Mariam Jamal-Hanjani, Nicola Steele, Salma Naheed, Craig Dick, Fiona Taylor, Helen Adderley, Fiona Black, Yvonne Summers, Matt Evans, Alexandra Rice, Aurelie Fabre, William A. Wallace, Siobhan Nicholson, Alex Haragan, Phillipe Taniere, Andrew G. Nicholson, Gavin Laing, Judith Cave, Martin D. Forster, Fiona Blackhall, John Gosney, Sanjay Popat, Keith M. Kerr

**Affiliations:** 1 Division of Molecular and Clinical Cancer Sciences, University of Manchester, Manchester, UK; 2 Department of Medical Oncology, The Christie NHS Foundation Trust, Manchester, UK; 3 Cancer Research UK Lung Cancer Centre of Excellence, London and Manchester, Manchester, UK; 4 Department of Histopathology, University Hospital Southampton NHS Foundation Trust, Southampton, UK; 5 Department of Cellular Pathology, University College London Hospital NHS Foundation Trust, London, UK; 6 Department of Histopathology, Papworth Hospital, Cambridge, UK; 7 Department of Medical Oncology, University College London Hospitals NHS Foundation Trust, London, UK; 8 Department of Medical Oncology, the Beatson West of Scotland Cancer Centre, Glasgow, UK; 9 Department of Medical Oncology, University Hospital Southampton NHS Foundation Trust, Southampton, UK; 10 Department of Pathology, Queen Elizabeth University Hospital, Glasgow, UK; 11 Department of Medical Oncology, Weston Park Cancer Centre, Sheffield, UK; 12 Department of Histopathology, the Newcastle Upon Tyne Hospitals NHS Foundation Trust, Newcastle, UK; 13 Department of Histopathology, Black Country Pathology Services, Royal Wolverhampton NHS Trust, Wolverhampton, UK; 14 Department of Histopathology, Royal Brompton and Harefield NHS Foundation Trust, London, UK; 15 Department of Histopathology, St Vincent’s Healthcare Group, Dublin, Ireland; 16 Department of Pathology, Royal Infirmary of Edinburgh, Edinburgh, UK; 17 Division of Pathology, the University of Edinburgh, Edinburgh, UK; 18 Department of Histopathology, St James’s Hospital, Dublin, Ireland; 19 Department of Cellular Pathology, Royal Liverpool University Hospital, Liverpool, UK; 20 Department of Histopathology, University Hospitals Birmingham NHS Foundation Trust, Birmingham, UK; 21 National Heart and Lung Institute, Imperial College, London, UK; 22 Department of Pathology, Aberdeen University School of Medicine and Aberdeen Royal Infirmary, Aberdeen, UK; 23 Department of Oncology, University College of London Hospital and UCL Cancer Institute, London, UK; 24 Lung Unit, The Royal Marsden Hospital, London, UK; 25 Section of Clinical Studies, The Institute of Cancer Research, London, UK

**Keywords:** Non-small-cell lung cancer, Non-small-cell lung cancer, Lung cancer

## Abstract

Over the past 10 years, lung cancer clinical and translational research has been characterised by exponential progress, exemplified by the introduction of molecularly targeted therapies, immunotherapy and chemo-immunotherapy combinations to stage III and IV non-small cell lung cancer. Along with squamous and small cell lung cancers, large cell neuroendocrine carcinoma (LCNEC) now represents an area of unmet need, particularly hampered by the lack of an encompassing pathological definition that can facilitate real-world and clinical trial progress. The steps we have proposed in this article represent an iterative and rational path forward towards clinical breakthroughs that can be modelled on success in other lung cancer pathologies.

Large cell neuroendocrine lung carcinoma (LCNEC) is a rare lung cancer subtype for which progress in management has fallen behind the significant developments seen in non-small cell lung cancer (NSCLC) using targeted therapies and checkpoint inhibitors.^1^ The World Health Organisation classify LCNEC as a neuroendocrine carcinoma, while its management and clinical phenotype have often been considered to be consistent with small cell lung carcinoma (SCLC).^2^ Two key clinical differences from SCLC are that (i) it is more likely to present with early stage disease (~25% of cases), and (ii) primary lesions are more likely to be peripheral.^3^ More in keeping with SCLC, patient survival at stage IV is generally poor (median < 1 year), a figure that represents a paucity of preclinical research and clinical trials in this area; a striking contrast to ALK-rearranged NSCLC, which represents a NSCLC subgroup of similar prevalence whose survival has been upgraded by several years as a consequence of recent remarkable progress with targeted therapy.^4–6^

This short review and consensus statement from the British Thoracic Oncology Group (BTOG) and the Association of Pulmonary Pathologists (APP) was produced through a process of iterative review amongst members. A skeletal first article draft suggesting main sub-headings was sent to APP and BTOG members in February 2020. Feedback was received from co-authors by April 2020, with re-draft based on their comments. All authors declared themselves agreed with this version in June 2020. Members were also asked to highlight key articles that may be missing from initial drafts.

Overall, we aim to (a) define the fundamental questions that have limited progress in LCNEC to date, and (b) set out a framework for the pursuit of translational and clinical progress in the years to follow. At the heart of this ambition is a determination to resolve the following fundamental questions:

1. What is large cell neuroendocrine lung carcinoma?
2. How many patients are affected by it?
3. What is standard of care treatment?

Unless these questions are answered conclusively, clinical progress for patients with this cancer will continue to be limited.

We will finish by describing a LCNEC ‘agenda for change’ to answer these questions, including plans for a blinded review of historical cases and a common registry of all LCNEC patients. It is hoped that this approach will set solid foundations from which LCNEC clinical trial proposals and treatment breakthroughs emerge for future patient benefit.

## What is large cell neuroendocrine lung carcinoma?

Although neuroendocrine differentiation in lung tumours has been described in several papers since the 1970s, defining criteria were first proposed in 1991.^7^ These have essentially remained the same to the present day (WHO criteria). LCNEC has been classified as a neuroendocrine carcinoma that appears to confer a SCLC clinical phenotype.^2^ Its diagnostic challenges, especially in small biopsy material, have presented a substantial problem to pathologists over several years, creating a clinical context where there is little confidence that LCNEC is being consistently labelled as the same disease. This variability can be considered in terms of both traditional histopathological assessment as well as modern molecular pathological analysis, and is threatened even further by an increasing emphasis on diagnostic optimisation using limited pathological and cytological samples.^8, 9^

### Histopathology

Although small biopsies and EBUS cytology specimens provide ~85% of lung cancer diagnoses in the UK and the majority of cases internationally, many pathologists feel they are unable to make a definitive LCNEC diagnosis without examination of a resection specimen. Indeed, existing WHO LCNEC classification cautions against definitive diagnosis unless a very substantial excision biopsy or resection is available. The difficulties posed by the histological classification are exemplified in two phase 2 clinical trials that have focused on LCNEC, both of which reclassified ~25% of recruited patients to an alternative SCLC or NSCLC diagnosis following central pathological review.^10, 11^

**Fig. 1:**
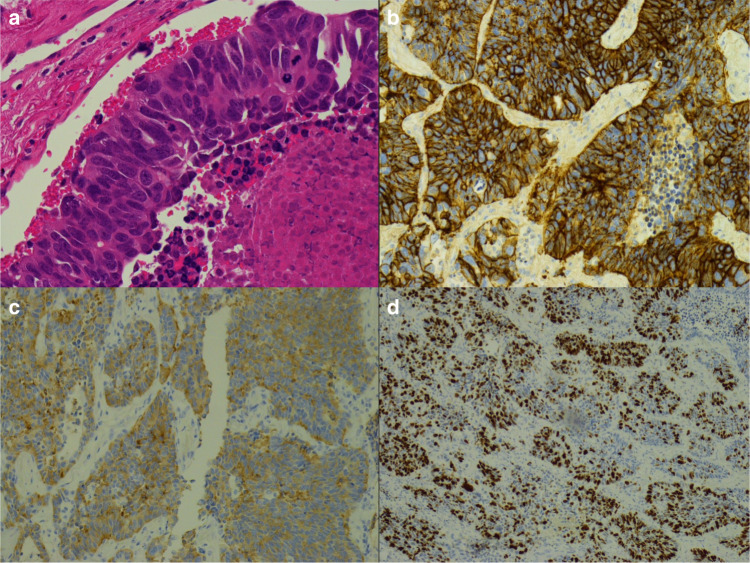
Large cell neuroendocrine carcinoma. **a** Morphology showing cytoplasmic abundance, the presence of nucleoli and nuclear size (haematoxylin and eosin, ×400). **b**–**d** immunohistochemistry: **b** CD56 (×200); **c** synaptophysin (×100); **d** Ki67 (×100).

Current WHO criteria for LCNEC diagnosis include (i) neuroendocrine morphology (trabeculae, palisading, organoid nesting and/or rosette formation), (ii) high proliferation rate (>10 mitoses per 10 high-power fields), (iii) extensive geographic necrosis and (iv) IHC expression of at least one neuroendocrine marker (chromogranin-A, synaptophysin, NCAM/CD56) (Fig. 1). The challenge of making this diagnosis is further complicated by the fact that these criteria are used as a classification for two different patterns of carcinoma. First, high-grade NSCLC showing morphological evidence of neuroendocrine differentiation and expressing neuroendocrine markers. Second, lower-grade tumours resemble an atypical carcinoid (AC) tumour, which, on closer inspection, have a mitotic count of >10/2 mm^2^.

**Fig. 2:**
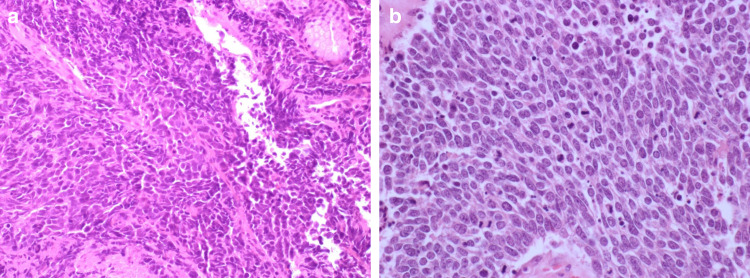
Architectural quality of biopsy versus resection specimens. **a** Small cell lung cancer biopsy showing crushing and loss of architecture (haematoxylin and eosin, ×200). **b** Small cell lung cancer resection sample (haematoxylin and eosin, ×400). Images provided by Dr Anna Paterson.

LCNEC is distinguished from SCLC via cytomorphological assessment of cytoplasmic abundance, the presence of nucleoli and nuclear size (Fig. 1).^7^ Assessment and interpretation of these criteria may be difficult with cytology specimens or small biopsies that can be crushed and morphologically heterogeneous, lacking diagnostic architectural features (Fig. 2). Commonly samples are labelled using alternative descriptive terms such as ‘combined NSCLC/SCLC’, ‘NSCLC with neuroendocrine differentiation’ or ‘high grade neuroendocrine carcinoma’ rather than LCNEC, so standardisation of this nomenclature will be of paramount importance for progress. Parallel experience from the Dutch pathology registry (PALGA) has suggested that an LCNEC diagnosis from biopsy requires (i) NSCLC morphology devoid of squamous or adenocarcinoma features, and (ii) positive staining with IHC of ≥2 neuroendocrine markers (Fig. 1).^12^ This contrasts with existing criteria from the WHO that recommends only ‘non-small cell carcinoma, with neuroendocrine morphology and immunophenotype, possible LCNEC'.^13^

In order to empower pathologists to facilitate future clinical progress, we must (a) clarify what sample type is mandatory to make a confident diagnosis, (b) define a common ‘one-size fits all’ diagnostic label for LCNEC that either excludes/includes cases of ‘combined SCLC/NSCLC’ and ‘NSCLC with neuroendocrine differentiation' or (c) provide a diagnostic categorisation which can embrace several potential diagnostic groups, including those where there is diagnostic doubt. One way to support a definition may be to identify if overlapping molecular pathology exists in cases that are difficult to label. Table 1 offers further speculation on how LCNEC diagnostic criteria may develop over the coming 1–2 years. Central to these changes will be a supportive MDT environment that harmonises the different elements of LCNEC histology, imaging and presentation, facilitating further biopsy and/or tumour resection when it is considered appropriate and in the patient’s best interests. There now also exists an opportunity to revisit the conventional histopathological diagnostic criteria for LCNEC, refining its diagnosis using a combination of morphological, immunohistochemical and molecular criteria.

**Table 1:** Possible future criteria for LCNEC definition.

Resection samples^a^	Small biopsy samples^a^
1. LCNEC (+/− combined adenocarcinoma +/− combined SCLC). 2. LC carcinoma with NE differentiation. 3. LC carcinoma with NE morphology.	1. LCNEC (only rare cases with sufficient sample). 2. ‘Probable’ LCNEC, NSCLC + NE morphology and/or NE markers. 3. High grade NE carcinoma (HGNEC) NOS (i.e. could be SCLC or LCNEC).

*LCNEC* large cell neuroendocrine carcinoma, *LC* large cell, *NE* neuroendocrine, *NOS* not otherwise specified, *SCLC* small cell lung cancer, *NSCC* non-small cell carcinoma.

^a^Where possible, Rb1/p16 IHC and targeted NGS to be performed for the purpose of further prospective scrutiny.

### Molecular pathology

Following on from a cluster of initial reports that were limited by the patient number and/or scope of analysis,^14–18^ three sequencing studies have offered large-scale molecular characterisation of LCNECs, the methods and results of which are summarised in Table 2.^19–21^ Methodological similarities between these studies include relatively small patient numbers (range 45-78) and concomitant sequencing/analysis of SCLC as a control cohort. The most striking mutational results suggested a clear division of LCNEC into ‘NSCLC-type’ and ‘SCLC-type’, a sub-classification that could be made on the basis of *RB1* and/or *KRAS* and/or *STK11/LKB1* mutation. These mutations have been frequently identified in the Cancer Genome Atlas and other large-scale genomic analyses as genetic hallmarks of NSCLC or SCLC.^22^ On the face of it, these findings may corroborate the perception of LCNEC as a ‘trans-differentiation’ phenomenon, implying that a second biopsy at a spatially distinct region of the same tumour would clarify a definitive NSCLC versus SCLC histology. However, what emerged in the largest and most detailed sequencing study was a transcriptional taxonomy suggestive that LCNEC is a separate cancer entity, rather than a continuum of existing lung cancer classifications; one that is closely aligned with SCLC, but also featuring novel expression profiles and higher levels of tumour mutational burden (TMB) than that seen in NSCLC or SCLC.^21^

**Table 2:** Molecular characterisation of LCNEC.

Study	No. of patients	Methods	Genomic data	Control comparator
Rekhtman et al.^19^	45	NGS (MSK-IMPACT) of paired LCNEC tumour/normal tissue Control: other lung carcinoma histologies (*n* = 242 ADC = 151, SqCC = 36, SCLC = 42, carcinoids = 13)	SCLC-like LCNEC (*n* = 18), *TP53* + *RB1* co-mutation/loss, absence *STK11 and KRAS*, evidence *MYCL* amplification, *SOX2* + *FGFR1* NSCLC-like LCNEC (*n* = 25), *STK11, KRAS, KEAP1* mutations + lack of *TP53* + *RB1* co-mutation/loss Carcinoid like (*n* = 2), *MEN1* mutation + low mutational burden	SCLC-like vs. SCLC: *KEAP1* 33% vs. 5% (*P* = 0.008) NSCLC-like vs. ADC: *STK11* 60% vs. 16% (*P* = 0.0001), *NOTCH1-4* 28% vs. 7% (*P* = 0.005), *EGFR* mutation: 0% vs. 25% (*P* = 0.0002), co-mutation *STK11* + *TP53*: 40% vs. 8% (*P* = 0.0002)
George et al.^20^	75	WES + /− WGS: genomic analysis (*n* = 60) Transcriptomic analysis (*n* = 69) Control: SCLC	Type I LCNEC (37%)*: TP53* + *STK11/KEAP1* mutations *ASCL1*^high^, *DLL3*^high^, *NOTCH*^low^ Type II LCNEC (42%): *TP53* + *RB1* mutation *ASCL1*^low^, *DLL3*^low^, *NOTCH*^high^	SCLC: *TP53* + *RB1* mutation *ASCL1*^high^, *DLL3*^high^, *NOTCH*^low^
Miyoshi et al.^21^	78	NGS: targeted capture sequencing coding on exons of 244 cancer genes Control: SCLC (*n* = 141)	Altered genes LCNEC: *TP53* (71%), *RB1* (26%), *KRAS* (6%), *FGFR* (5%), *KIT* (4%), *ERBB2* (4%), *HRAS* (1%), *EGFR* (1%) LCNEC: alterations PI3K/AKT/mTOR pathway (15%)	LCNEC vs. SCLC: *RB1* 26% vs. 40% (*P* = 0.039) LCNEC vs. SCLC: alterations PI3K/AKT/mTOR pathway 15% vs. 17%

*NGS* next generation sequencing, *MSK-IMPACT* Memorial Sloan Kettering-Integrated Mutation Profiling of Actionable Cancer Targets, *LCNEC* large cell neuroendocrine cancer, *ADC* adenocarcinoma, *SqCC* squamous cell cancer, *SCLC* small cell lung cancer, *WES* whole exome sequencing, *WGS* whole genome sequencing.

An important consideration with all this work is to avoid the assumption that the presence of *RB1* mutation or loss confirms a SCLC phenotype—its contribution to NSCLC has been well characterised.^23^ There also remains a strong argument that three additional and well established immunohistochemical tests (RB1, p16, LKB1) may suffice to categorise LCNEC into ‘SCLC-type’ and ‘NSCLC-type’ spectrums, should these sub-classifications be deemed suitable for further clinical trial interrogation.^24, 25^ Moreover, reports of prolonged responses to tyrosine kinase inhibitors in LCNEC harbouring *EGFR* mutations or *ALK* rearrangements suggest that standard of care molecular testing can still offer significant benefits to a finite group of patients^26–28^; whether these cases represent those LCNECs that are mixed with adenocarcinoma is difficult to establish. Molecular overlap of ACs and LCNECs has also been identified, whereby a small number of tumours with histological features of AC can, in particular, adopt *TP53* +/− *RB1* mutations that are associated with poorer outcomes.^29–31^ Finally, those involved in standardisation of the future diagnostic process must closely consider which sequencing techniques will be feasible with small samples and require integration into molecular pathology labs. The current crop of data suggests that exome and transcriptomic sequencing must at least be considered, although it is unclear at this point if this will help resolve differences or confuse things further.

## How many patients are affected by LCNEC?

An implicit aspect of answering this question definitively will be a resolution to question one above: a conclusive set of LCNEC diagnostic criteria. Thus, we can only broadly estimate LCNEC patient numbers at present, and there currently exists no data capture of LCNEC cases in the UK National Lung Cancer Audit (NLCA). Existing international data suggest it accounts for 2–3% of lung cancer cases, a considerable number of patients given the high global incidence of lung cancer.^2, 32^ With a projected 46,000 new cases of lung cancer cases in the UK per annum, we estimate that ~1000–1500 patients could be diagnosed with LCNEC each year, ~75% of whom will present at an advanced stage. For ~229,000 new lung cancer cases per annum in the US, we estimate 4500–7000 will be diagnosed with LCNEC. Thus, there exists no good reason, as far as patient numbers are concerned, to consider recruitment to LCNEC clinical trials as ‘unachievable’. By comparison, *ALK*-rearranged NSCLC patients represent a similar sized lung cancer subgroup and have been the basis of pioneering international randomised phase 3 trials involving up to 343 patients.^4–6^ The main contrast between LCNEC and ALK subgroups is that the latter currently represents an easily identifiable subtype, readily diagnosed using small biopsies. Diagnostic uncertainty also means we are likely underestimating the true percentage of LCNEC cases, and there may be unestablished disparities in LCNEC incidence between early and advanced disease. Using the example of ALK as a paradigm for ‘rare’ lung cancer clinical research, potential opportunities for further trial development in LCNEC will be the International Rare Cancer Initiative (IRCI) affiliated with the UK National Institute for Health Research (NIHR) and Cancer Research UK, the European Reference Networks (ERNs) created to deal with rare tumours, and the European Organisation for Research and Treatment of Cancer (EORTC) which can be used as a platform for data registration and clinical studies.

## What is the standard of care treatment?

As with question 2, a complete answer to this question will ultimately follow on from a definitive LCNEC definition. For our day-to-day practice, the lung cancer community will remain unclear on the best choice of routine treatment unless there is an acceptable and reproducible pathological consensus on criteria for the classification of LCNEC in small diagnostic biopsy and cytology samples. If it is concluded that LCNEC is separate from other subtypes of NSCLC or SCLC, it can also be reasonably argued that no standard of care (SOC) exists due to an absence of phase 3 and/or randomised clinical trials. In turn, there would be a justifiable niche for first line clinical trial proposals given the very modest clinical benefits that have been observed in the prospective setting using chemotherapy.

In view of these data limitations, an informative body of retrospective and real-world data has been accumulated over a number of years by colleagues in the US and the Netherlands. In early stage disease, surgical resection is the preferred treatment,^33^ with results from over 6000 stage I–IIIA patients included in the US National Cancer Database demonstrating superior survival vs. stereotactic radiation or chemoradiotherapy.^34^ Review of 1672 patients included in this database suggested LCNEC is the only subtype of NSCLC which could benefit from adjuvant chemotherapy at all operable stages (IA–IIIA).^35^ In its advanced stages, experience from the Dutch PALGA network have suggested that NSCLC-based combinations (platinum-gemcitabine/taxanes) may perform better than traditional platinum-etoposide approaches, particularly in ‘NSCLC-type’ cases expressing wild-type Rb1.^25, 36^ Such an approach suggests LCNEC could be optimised by a precision medicine optimisation for treatment stratification, whether it be with novel agents or traditional chemotherapies.

Existing/limited prospective clinical trial evidence includes two prospective single arm phase 2 trials examining the combination of cisplatin-etoposide or cisplatin-irinotecan in 42 and 44 advanced-stage patients, respectively.^10, 11^ Median overall survival (OS) with these treatments was 7.7 months (95% CI, 6–9.6) vs.15.1 months (95% CI, 11.2–19), respectively. After a centralised pathology review of 41 patients who received cisplatin-irinotecan, 10 were reclassified to SCLC and 1 to NSCLC. Median survival time was 12.6 months (95% CI, 9.3–16.0) in the LCNEC group vs. 17.3 months (95% CI, 11.2–23.3) in the SCLC group (*P* = 0.047). When combined ~25% of patients in both studies were reclassified to an alternative diagnosis (mostly SCLC) following central pathological review. More recently, the addition of everolimus to paclitaxel and carboplatin in a phase 2 study of 49 LCNEC patients led to a median OS of 9.9 months and a 51% incidence of grade 3–4 toxicities.^37^

A final inescapable question in the current NSCLC treatment landscape is whether LCNEC could be vulnerable to treatment with immune checkpoint inhibitors (ICIs). Despite being characterised as having low levels of PD-L1 positivity, preliminary data from the US have suggested response rates to ICIs are perhaps above what might have been expected for a low PD-L1 cancer, particularly in ‘SCLC-type’ disease.^38^ Given the high TMB suggested, LCNEC may yet represent a relatively unique cancer histology where TMB proves far more informative than PD-L1 IHC for prediction of ICI benefit.^19, 39^ It should however be noted that the role of TMB as a biomarker for ICI remains a contentious one, with studies including CheckMate-227 identifying that it may not be predictive of survival benefit.^39^ Of LCNEC patients who are resistant to ICIs, one might predict that their cancers are ‘NSCLC-type’ LCNEC harbouring STK11 mutation, a mutation in NSCLC which has been strongly associated with ICI resistance.^40^ A number of key questions will require UK, European and international consensus on treatment strategy once pathological criteria have been agreed, for instance (i) should we offer SCLC-based chemotherapy if *RB1* mutation or loss is established, and (ii) should we also offer prophylactic cranial irradiation to these cases?

## Forging a path forward

Below we propose a series of practical steps for optimising management and treatment approaches in LCNEC, with a view to establishing foundations for further clinical trial work.

1. Establishing an LCNEC definition
  i. Blinded inter-centre assessment of candidate LCNECs. A network of APP pathologists will receive tissue blocks from resection and biopsy samples for the following NSCLC classifications: LCNEC, SCLC, combined SCLC/ NSCLC, combined LCNEC/ NSCLC, ACs with high mitotic count and NSCLC with neuroendocrine morphology (+/− NE IHC positivity). Their analyses and interpretation of sections will be blinded, and they will be asked whether the sample is deemed to be of sufficient quality for making a definitive diagnosis. Where possible, paired samples (biopsy/resection) will be assessed. Targeted sequencing results for each sample will be made available following initial histopathological review, with each pathologist being asked if they would revise any of their conclusions on the basis of this updated molecular detail. All work will be preceded by a consensus choice of nomenclature and an agreed strategy for interpreting molecular data.
  ii. Spatial heterogeneity. A small number of LCNECs have been sequenced in the Cancer Research UK (CRUK) TRACERx programme,^41^ offering the potential to assess whether LCNEC represents a trans-differentiation state between NSCLC and SCLC. Exomic profiles will be examined from spatially distinct regions of resected tumours. The postmortem PEACE study and recurrence samples from TRACERx represent a possible further opportunity to detail these changes. An anticipated small number of samples means that it will be challenging to validate any insight gained in the more common setting of advanced LCNEC, leaving this exploration more likely to remain in the realm of discovery rather than translational research.
    Overall, completion of this work should be facilitated by the recent roll-out of a common infrastructure for molecular testing in NHS England, with a drive towards routine next generation sequencing coinciding with the creation of seven genomics laboratory hubs across England.
2. Establishing the true incidence of LCNEC.
  All true LCNEC lung cases will be ultimately documented in the National Lung Cancer Audit (NLCA).^42^ Depending on the results of the work proposed above, this definition may be revised following completion of multi-site pathology review.
3. Establishing standard of care treatment and facilitating future clinical trials.
  i. An up-to-date anonymised registry of LCNEC cases will be compiled and updated, documenting pathological, immunohistochemical, surgical resection status (R0, R1, R2, R uncertain), systemic treatment, morbidity, relapse-free survival and overall survival details. Quarterly TCs will review challenging cases from this series. Retrospective outcomes in early and advanced disease will be assessed after a sufficient period of follow-up.
  ii. For advanced stage, stratification of chemotherapy using Rb1 and p16 immunohistochemistry should be considered, in line with the approach described by the Netherlands.
  iii. On the basis of previously described US data, adjuvant treatment should be considered in all LCNEC patients following resection.^35^ Choice of chemotherapy in this setting will require consensus, but may also be stratified according to Rb1 and p16 status. Given there is firmer ground for diagnosing LCNEC using resection tissue, it is feasible to consider a clinical trial of adjuvant chemotherapy for stage I disease.
  iv. In the medium-term, use the foundations of the work proposed above as a renewed basis for clinical trial proposals in late stage disease. Similar to recent breakthroughs in targeting KRAS-mutant NSCLC, emerging knowledge in the treatment of LCNEC offers the possibility for a significant impact on patient care.^43^

## Conclusions

The steps we have proposed in this article represent an iterative and rational path forward towards clinical breakthroughs that can be modelled on success in other lung cancer pathologies. Progress will likely be measured in 3–5-year intervals given there are no quick solutions to some of the difficulties discussed. However, the appetite amongst the lung cancer community to remedy this unmet need remains without question.

## Data Availability

Not applicable.
